# Evidence for Rab7b and Its Splice Isoforms Having Distinct Biological Functions from Rab7a

**DOI:** 10.3390/ijms26062610

**Published:** 2025-03-14

**Authors:** Wing Hei Wong, Stephanie Z. Liu, Annie Shi Ru Li, Xingyou Liu, Morris F. Manolson, Ralph A. Zirngibl

**Affiliations:** 1 Faculty of Dentistry, University of Toronto, Toronto, ON M5G 1G6, Canada; stephanieziyue.liu@mail.utoronto.ca (S.Z.L.); annieshiru.li@mail.utoronto.ca (A.S.R.L.); xingyou.liu@alumni.utoronto.ca (X.L.); ralph.zirngibl@utoronto.ca (R.A.Z.); 2 Department of Medical Biophysics, University of Toronto, Toronto, ON M5G 2C4, Canada

**Keywords:** Rabs, Rab7, Rab7a, Rab7b, Rab7 nucleotide/point mutants, Rab7 splice isoforms, alternative splicing

## Abstract

The Rab family of small guanosine triphosphatases (GTPases) are nucleotide-dependent switches. Mutations in Rabs can result in human diseases. Rab7a and Rab7b transition from early endosomes to lysosomes and are presumed to function similarly. Most studies look at Rab7a, less on Rab7b, with the underlying assumption they function similarly. There have yet to be articles comparing them side by side. Whilst cloning Rab7 homologues, we identified splice isoforms for Rab7b only. These splice isoforms, Rab7b2 and Rab7bx8 lacking different exons, have not been previously characterized but suggest alternative function(s) for Rab7b. Thus, we hypothesize that Rab7 homologues have distinct functions. Here, we compare Rab7a and Rab7b nucleotide mutants locked in GDP-bound (Rab7T22N), GTP-bound (Rab7Q67L), nucleotide-free (Rab7aN125I/Rab7bN124I) states and characterized localization of the Rab7b splice isoforms. HeLa cells were transiently transfected with fluorescently tagged Rab7 reporters. Confocal images were processed with ImageJ and analyzed with SPSS. Rab7a and Rab7b nucleotide mutants were significantly different to one another. Approximately 50% of Rab7b splice isoform-expressing cells had aggregated vesicles, which were phenotypically different from Rab7b vesicles. Rab7a and Rab7b vesicles shared approximately 60% colocalization with each other, while Rab7b vesicles preferentially localized to the Trans Golgi Network. Our results suggest Rab7b is distinct from Rab7a, and Rab7b splice isoforms have different biological functions.

## 1. Introduction

**Figure 1:**
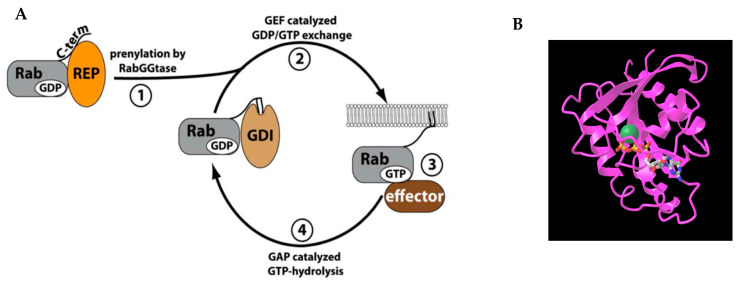
(**A**). Rab cycle: 1. Rabs are prenylated by geranylgeranylation of two cysteines at the C-terminus. 2. Recruitment of GEF for binding of GTP with Rabs. 3. GTP-bound Rabs can bind with effector molecules for vesicular trafficking. 4. Rabs are inactivated by GAPs through GTP hydrolysis, producing GDP-bound Rabs [2]. (**B**). Crystal structure of Rab7a comprised of six β-sheets with four α-helices (PDB: 1T91) [6].

**Figure 2:**
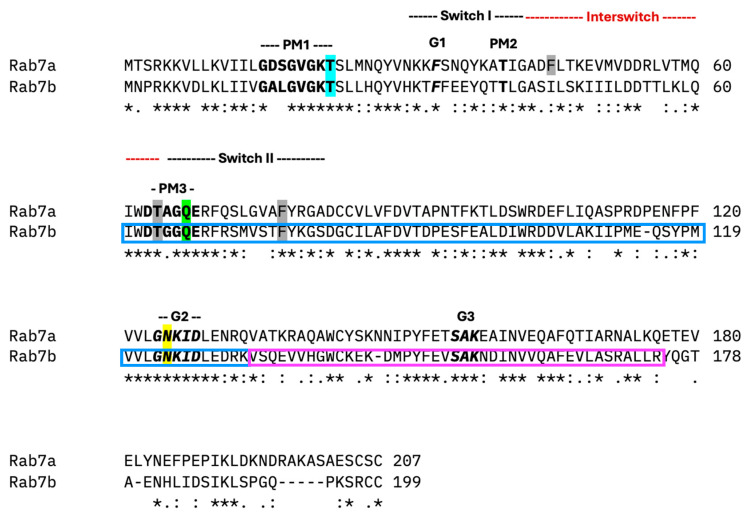
Clustal alignment of mouse Rab7a and Rab7b [27]. Regions sharing the same amino acid in all sequences are depicted with asterisks (*). Amino acids with similar and less similar properties are depicted by colons (:) and dots (.) respectively. The highlighted motifs are representative of the three nucleotide mutants: T22N, Q67L, N125I/N124I in blue, green and yellow, respectively. The conserved PM1-3 motifs (bold) and G1-3 motifs (bold and italicized) and hydrophobic triad are highlighted in grey. The missing exons of Rab7b2 and Rab7bx8 are represented by the magenta and blue boxes, respectively.

Rab proteins are small GTPases that switch between a GTP-bound ‘ON’ and GDP-bound ‘OFF’ state facilitated by guanine nucleotide exchange factors (GEF) and GTPase-activating proteins (GAP) [1,2] (Figure 1A). Rabs facilitate lipid vesicle movement, tethering, and membrane fusion. The process of Rab membrane targeting requires the prenylation of cysteine(s) at the C-terminus and a GEF on the target membrane. The GEF allows GTP to bind with the Rab promoting effector molecule binding and packaging into vesicles for transport/fusion with compartments. Effector molecules interact with the switch regions of Rabs. These switch regions undergo conformational changes: flexible conformations in GDP-bound Rabs and effector-specific conformations in GTP-bound Rabs. GTP hydrolysis completes the Rab cycle and recycles it to the donor compartment via GDP-dissociation inhibitor (GDI) [3,4]. All Rab proteins recognize effectors through their conserved regions: Switch I (SWI), Switch II (SWII), and Interswitch region [5] (Figure 2). The nucleotide-binding site is surrounded by conserved motifs consisting of three phosphate or magnesium binding motifs (PM1–3) and three guanine-binding motifs (G1–3) [5] (Figure 2). Hydrophobic patches on Rabs, consisting of three conserved aromatic residues and non-polar residues of the Switch I and II regions, interact with a complementary hydrophobic region on effector proteins (Figure 2) [4]. All Rabs have a conserved GTPase protein fold that consists of six β-sheets with four to five surrounding α-helices (Figure 1B) [6,7]. These motifs are recognized by proteins such as GEFs or GAPs to interact with the Rab and facilitate its function [8]. The hypervariable C-terminal domain of Rab proteins contains structural determinants for the specific subcellular localizations of some Rab proteins with their effector membranes [5,9]. Localization of Rab proteins is not only determined by its hypervariable C-terminal domain but also due to interactions with other effectors and accessory proteins for targeting into specific compartments [10,11].

Rab7 functions in cellular trafficking [12]. There are two homologues of Rab7: Rab7a and Rab7b. It is unclear if the Rab7 homologues share similar functions or are distinct from one another. Rab7a, the well-studied homologue, functions in the regulation of early to late endosomal maturation and the transport and fusion of late endosomes with lysosomes in the periphery of the nucleus [12]. Rab7a regulates autophagolysosomal fusions, cellular trafficking and degradation, and the interaction between lysosomes and mitochondria [13]. Rab7b regulates endosomal and Trans Golgi Network (TGN) transport, recycling to the TGN, and negative regulation of inflammatory responses [2]. Rab7a and Rab7b are assumed to have similar functions due to shared localization to the late endosomes and lysosomes, evidence that Rab7a is found at the TGN, and a shared GAP protein TBC1D5 [12,14,15].

However, there are several articles characterizing properties that are specific to either Rab7a or Rab7b, not both [12,16,17]. The movement and transport of Rab7a is facilitated through its effector Rab Interacting Lysosomal Protein (RILP) upon binding with a dynein motor [12]. RILP is not found to be associated with Rab7b [2]. Additionally, the movement of Rab7b is facilitated through a separate motor, myosin II, on the actin cytoskeleton [18]. Other functional differences between the Rab7 homologues are observed in regulation of oligodendroglial cell differentiation [17] and regulation of autophagy [19]. Physiologically, knockouts of Rab7a in mice are embryonically lethal, and missense mutations of Rab7a are associated with Charcot–Marie–Tooth Type 2B disease (CMT2B) [20,21]. A number of articles demonstrate the role of Rab7b in hemolytic uremic syndrome and Pelizaeus–Merzbacher disease, suggesting the absence of Rab7b is beneficial in disease recovery [17,22,23]. Together, the evidence suggests that the Rab7 homologues are different.

The shared and distinct properties between the Rab7 homologues are well reported, but there has been no comparison between Rab7a and Rab7b. We compare the Rab7 homologues under the same experimental conditions to determine similarities and differences in their functions.

Two splice isoforms of Rab7b were isolated while cloning full length Rab7b. Rab7b2 skips Exon 8 and Rab7bx8 skips Exon 7. The two splice isoforms are both expressed in multiple mouse tissues, and mRab7bx8 is expressed during the early stages of osteoclastogenesis. The production of splice isoforms is specific to Rab7b and was not seen in Rab7a. Therefore, we hypothesize that Rab7b splice isoforms have significant biological functions. Here, we aim to characterize any differential localization and vesicle size in Rab7b splice isoforms which could provide information regarding its functions.

The well-described Rab7a and Rab7b nucleotide mutants, Rab7a/bT22N (Rab7a/bT), Rab7a/bQ67L (Rab7a/bQ), and Rab7a125I/b124I (Rab7a/bN), are used to compare Rab7 homologues. The Rab7a/bT mutant has a threonine to asparagine substitution mutation in the GXXXGK(T) nucleotide-binding PM1 motif, affecting GTP affinity, and locking it in the GDP-bound form, thus acting as the dominant negative mutant (Figure 2) [24,25,26,27]. The Rab7a/bQ mutant has a glutamate to leucine substitution mutation in the DXXGQ motif of the SWII domain that is required for GTP hydrolysis, locking it in the GTP-bound form, thus acting as the constitutively active mutant (Figure 2) [25,26]. Rab7a/bN has an asparagine to isoleucine substitution mutation, which hinders the ability to bind with guanine nucleotides [24,25]. These mutations are expected to disperse the dominant negative Rab7a/bT and nucleotide-free Rab7a/bN in the cytoplasm while the constitutively active Rab7a/bQ is membrane-bound.

Overall, we aim to characterize the functional differences between Rab7a and Rab7b, including the localization and vesicle size of Rab7b splice isoforms.

## 2. Results

### 2.1. Clustal Alignment of Rab7a, Rab7b, and Its Splice Isoforms

The protein sequences of Rab7a and Rab7b in mice show 47.34% shared similarity and 62.8% shared identity [28]. The clustal alignments of Rab7a and Rab7b highlight the altered motifs in the nucleotide mutants (Figure 2). The spliced-out exons are indicated in Rab7b2 by the magenta box containing the G3 motif, and in Rab7bx8 by the blue box containing the PM3 and G2 motifs (Figure 2). The loss of these motifs could hinder the binding of nucleotides, potentially making Rab7b2 and Rab7bx8 phenocopy the nucleotide-free mutant. Rab7b2 is predicted to be able to bind effectors and be transported to vesicles as it is not missing the SWI and SWII regions. The absence of SWII region in Rab7bx8 may hinder effector binding and it is predicted that Rab7bx8 could alter vesicle trafficking.

### 2.2. Rab7 Homologues Have Different Morphologies and Localisation

**Figure 3:**
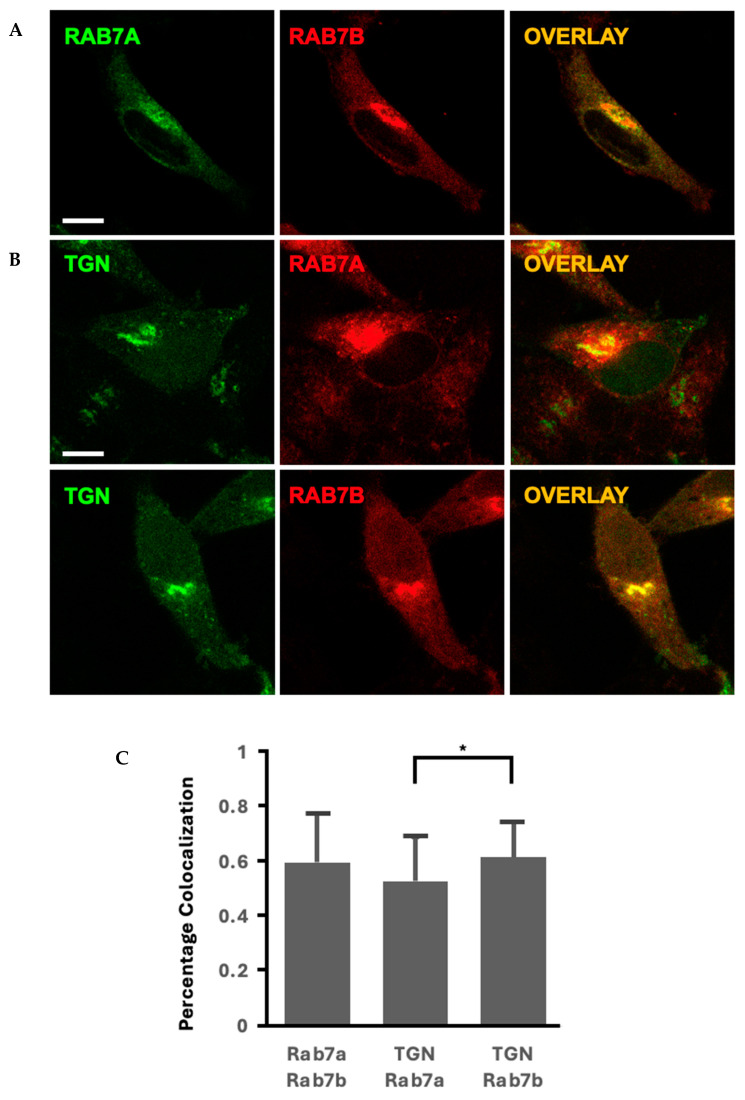
(**A**). Co-transfected Rab7a (GFP) with Rab7b (mCherry). Overlay (right column) demonstrates colocalization. Scale bar = 10 µm. (**B**). Co-transfected TGN46 (GFP) with Rab7a and Rab7b (mCherry). Overlay (right column) demonstrates colocalization. Scale bar = 10 µm. (**C**). Quantification of percentage of colocalization between the double transfections. Error bars represent the standard deviation. * *p* < 0.05.

Rab7a and Rab7b were co-transfected in HeLa cells, and transfections were imaged with confocal microscopy (Figure 3A). There was a 60% colocalization between the Rab7 homologues with portions of their vesicles in separate compartments (Figure 3A,C). Rab7a vesicles displayed a punctate morphology whilst Rab7b vesicles organized in a continuous linear structure (Figure 3A). The differences in vesicle morphology and localization suggest that the Rab7 homologues may be functionally distinct (Figure 3A).

The current literature states that the key difference between the Rab7 homologues is that Rab7b localizes to the TGN and is critical in TGN transport [29], but there are no papers that compare Rab7a and Rab7b localization to the TGN side by side. TGN46, a marker for the TGN described in the current literature, was used to identify colocalization between Rab7a and Rab7b (Figure 3B). We found that there was significantly greater colocalization of TGN46 with Rab7b compared to Rab7a (Figure 3C and Figure S6). This suggests that Rab7a and Rab7b localize together at some compartments, but Rab7b preferentially localizes to the TGN and that there may be a difference in function between the homologues with Rab7b having a prominent role in directing cellular materials to the TGN.

### 2.3. Subcellular Localization of Rab7a and Its Nucleotide Mutants in HeLa Cells

**Figure 4:**
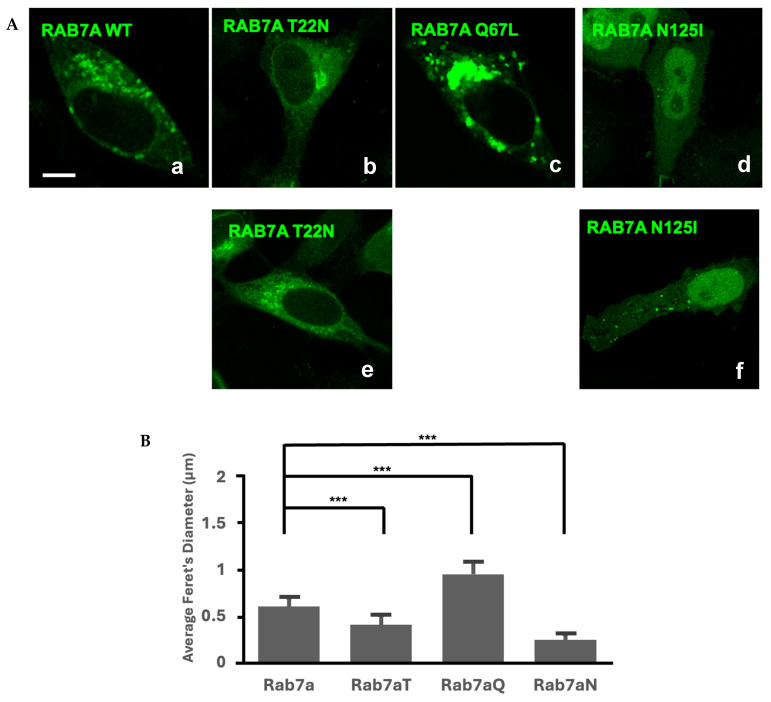
(**A**). Single transfected Rab7a and its nucleotide mutants in GFP. Scale bar = 10 µm. The panel is representative of the majority in the population (**a**–**d**) and the minority in the population (**e**,**f**). (**B**). Quantification of vesicle size (µm) of Rab7a compared against the nucleotide mutants, Rab7aT22N (Rab7aT), Rab7aQ67L (Rab7aQ), and Rab7aN125I (Rab7aN). Average vesicle size of Rab7a and mutants are represented by the bar graph. Error bars represent the standard deviation. *** *p* < 0.001. See Figure S3A for data analyses.

To characterize the differences in vesicle morphology and colocalization, Rab7a, Rab7b, and their nucleotide mutants’ vesicles were analyzed separately. Single transfections of Rab7a and its nucleotide mutants were imaged with confocal microscopy (Figure 4A,B). Images were captured from multiple experiments using both GFP and mCherry fluorescent tags. The data were combined as there was no statistically significant difference between the fluorescent markers (Figure S1A).

Rab7a localized to punctate vesicles spread across the cytoplasm with an average vesicle diameter of 0.60 µm (Figure 4(Aa)). The Rab7aQ67L mutant demonstrated similar localization to Rab7a, which is consistent with the current literature [25], but the vesicles were 58% larger than Rab7a and concentrated around the perinuclear region (Figure 4(Ac)). Approximately 80% of cells expressing the Rab7aT22N mutant had vesicles, which is contradictory to the current literature [30] (Figure 4(Ab)). On average, the diameter of these vesicles is 33% smaller than the diameter of vesicles from Rab7a. The majority of cells expressing the Rab7aN125I mutant did not contain vesicles and was cytoplasmic, in agreement with the literature [30] (Figure 4(Ad)). However, we consistently observed a minority cell population (<10%) that displayed small, well-defined vesicles with an average diameter 60% smaller than Rab7a, not previously mentioned in the literature [30] (Figure 4(Af)).

### 2.4. Subcellular Localization of Rab7b, Rab7b Nucleotide Mutants and Splice Isoforms in HeLa Cells

**Figure 5:**
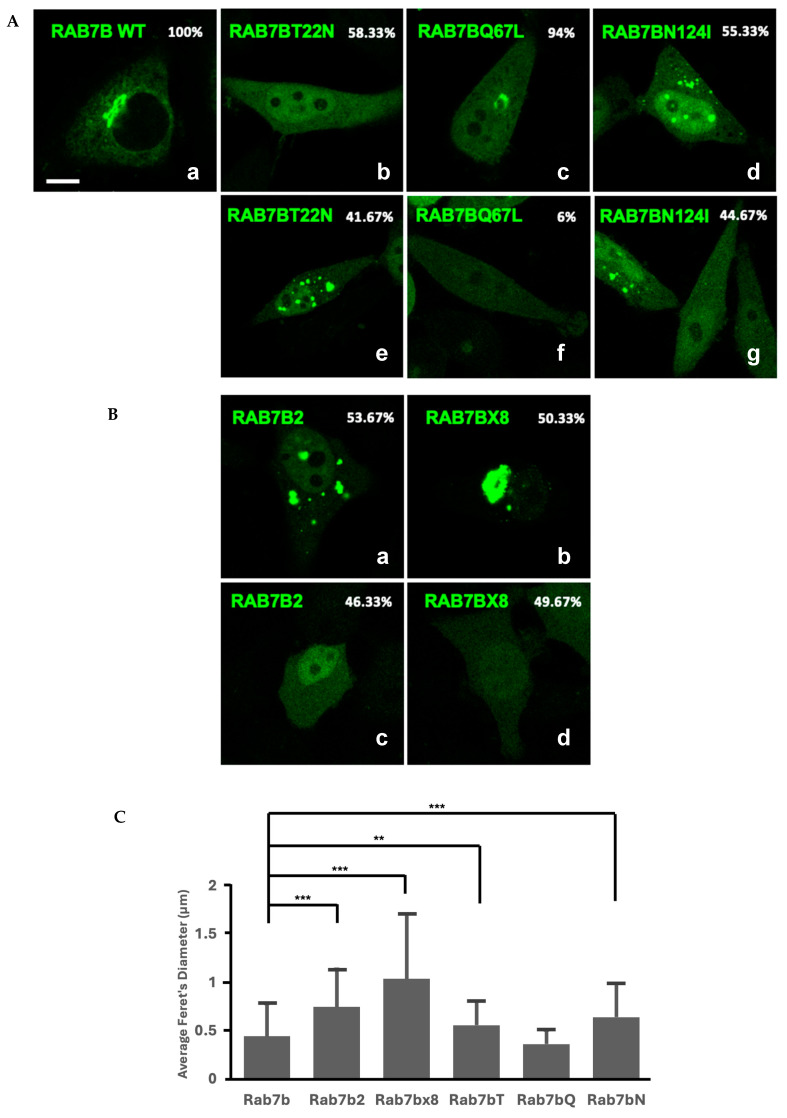
(**A**). Single transfected Rab7b and its nucleotide mutants in GFP. Scale bar = 10 µm. The panel is representative of the majority in the population (**a**–**d**) and the minority in the population (**e**–**g**). The percentage it represents is indicated on the top left corner. (**B**). Single transfected Rab7b splice isoforms in GFP. Scale bar = 10 µm. The panel is representative of the majority of the population (**a**,**b**) and the minority in the population (**c**,**d**). The percentage it represents is indicated on the top left corner. (**C**). Quantification of vesicle size (µm) of Rab7b compared against its splice isoforms (Rab7b2 and Rab7bx8), nucleotide mutants, Rab7bT22N (Rab7bT), Rab7bQ67L (Rab7bQ) and Rab7bN124I (Rab7bN). Average vesicle size of Rab7b and mutants are represented by the bar graph. Error bars represent the standard deviation. ** *p* < 0.01. *** *p* < 0.001. See Figure S4A for data analyses.

Single transfections of Rab7b, nucleotide mutants and splice isoforms were imaged with confocal microscopy Figure 5A,B. We observed two populations of cells in the case of the nucleotide mutants and the splice isoforms, with the majority population shown in the top row of Figure 5A,B.

Rab7b and Rab7bQ67L vesicles localized to a perinuclear region (Figure 5(Aa,c)) and showed similar vesicle morphology. The vesicle size for Rab7b was significantly less than Rab7bT22N and Rab7bN124I (Figure 5C). Rab7bQ67L demonstrated a similar perinuclear phenotype and vesicle morphology to Rab7b. A total of 58.33% of cells transfected with Rab7bT22N had no vesicles (Figure 5(Ab)). This phenotype is consistent with published literature where the Rab7bT22N dominant negative mutant demonstrated inhibition of lysosomal transport [30]. Similarly, 55.33% of cells transfected with Rab7bN124I displayed vesicles (Figure 5(Ad)).

The size of Rab7bQ67L vesicles were not significantly different to Rab7b (Figure 5C), with the average vesicle diameter 18% smaller than Rab7b, and this differs from Rab7a and Rab7aQ67L (Figure 5C). The remaining 41.67% of cells containing Rab7bT22N-positive vesicles were on average 26% larger than Rab7b (Figure 5(Ae),C). The cells expressing Rab7bN124I-positive vesicles were on average 44% larger than Rab7b (Figure 5C).

The size of Rab7b-positive vesicles was significantly smaller than the Rab7b splice isoforms (Figure 5C). The vesicle size of Rab7b2 and Rab7bx8 were 68% and 133% larger than Rab7b, respectively (Figure 5C). Cells transfected with Rab7b2 displayed vesicles that were spread out across the cytoplasm, and Rab7bx8, demonstrated large clusters of vesicles (Figure 5(Ba,b)). Furthermore, Rab7b vesicles were more elongated in shape as compared to the circular morphology of vesicles from the Rab7b splice isoforms (Figure 5(Ba,b)). When Rab7b was co-transfected with the splice isoforms, the splice isoform vesicles did not share the same localization with the Rab7b vesicles and appeared to be in distinct compartments (Figure S2). The difference in localization and vesicle size suggest that splice isoforms do not share the same function as Rab7b.

### 2.5. Nucleotide Mutants of Rab7a and Rab7b Cause Variations in Vesicular Size

**Figure 6:**
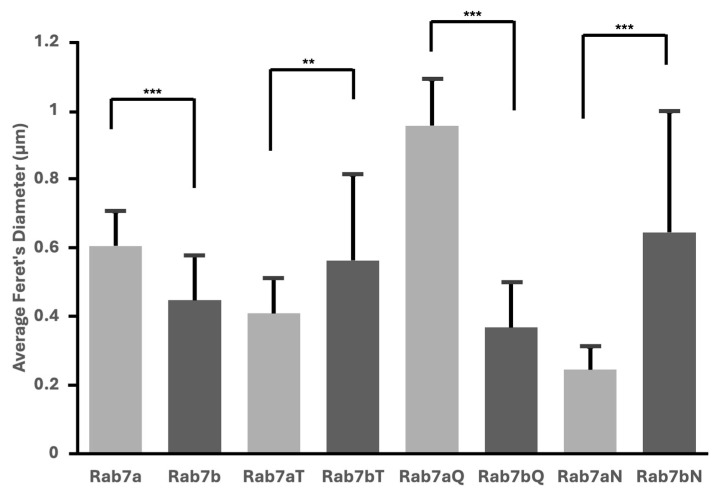
Quantification of vesicle size (µm) of Rab7a and its nucleotide mutants (Rab7aT, Rab7aQ, Rab7aN) compared to Rab7b and its nucleotide mutants (Rab7bT, Rab7bQ, Rab7bN). Average vesicle size of the Rab7a, Rab7b and their nucleotide mutants are represented by the bar graph. Error bars represent the standard deviation. ** *p* < 0.01. *** *p* < 0.001. See Figure S5 for data analysis.

Comparisons of Rab7a and Rab7b and their nucleotide mutants identified differences between the Rab7 homologues. The difference in vesicle size between Rab7a and Rab7b was statistically significant, and there were significant differences between all nucleotide mutants for the Rab7 homologues (Figure 6). The average Rab7bT22N and Rab7bN124I mutants were significantly larger than Rab7a equivalents, by 0.15 µm and 0.40 µm, respectively (Figure 6). The Rab7bQ67L was significantly smaller than Rab7aQ67L by 0.56 µm (Figure 6). These differences between Rab7a, Rab7b and their nucleotide mutants, which are representative of the different Rab cycle states, suggest that the roles of Rab7a and Rab7b are distinct.

### 2.6. Rab7a and Rab7b Nucleotide Mutants and Splice Isoforms Have Nuclear Vesicles

**Figure 7:**
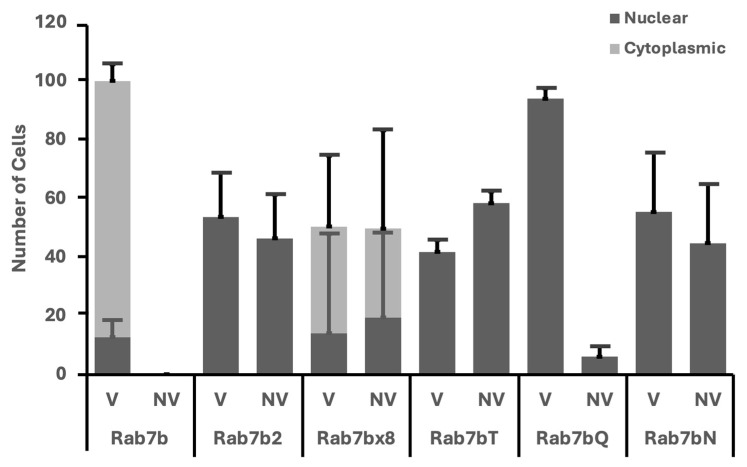
Categorization of the number of cells in Rab7b and its mutants presenting with the ‘vesicle’ (V) and ‘no vesicle’ (NV) phenotypes. Further categorization of those presenting with either phenotype with being ‘nuclear’ (dark grey) or ‘cytoplasmic’ (light grey). A hundred cells were counted for each mutant per experiment and averaged (shown in bar graph). Error bars represent the standard deviation.

A proportion of cells transfected with Rab7b, its nucleotide mutants and splice isoforms displayed a fluorescent signal in the nucleus, termed ‘nuclear’. The remainder of the cells with fluorescent signals in the cytoplasm were termed ‘cytoplasmic’. All Rab7b cells displayed vesicles with ~90% cytoplasmic and ~10% nuclear (Figure 7). In Rab7b2, the cell populations with and without vesicles were both nuclear. However, cells transfected with Rab7bx8 showed a combination of both nuclear and cytoplasmic in both the vesicle and no vesicle populations. There was more variability in Rab7bx8, demonstrated by the large error bars. All Rab7b nucleotide mutants are nuclear. Based on the observed nuclear phenotype in Rab7b mutants, we went back to analyze the Rab7a mutants to determine if the two homologues shared a similar nuclear phenotype. We found that Rab7a nucleotide mutants shared the nuclear phenotype, and this phenotype is currently being investigated.

## 3. Discussion

The conflicting consensus as to whether Rab7a and Rab7b are distinct from one another is due to a lack of direct comparisons under the same experimental conditions. The mechanisms and localizations of Rab7b and its splice isoforms have yet to be described in the literature.

### 3.1. Evidence That Rab7a and Rab7b Have Different Functions Based on Localization

The nucleotide mutants used in this experiment represent different stages of the Rab cycle that both Rab7a and Rab7b undergo, allowing us to analyze their localization and infer possible function.

The difference in vesicle size between Rab7a and Rab7b nucleotide mutants suggest that the homologues carry out differing functions in the active and inactive state (Figure 6). This may suggest that they function in different ways or associate with different effectors. Constitutively active Rab7aQ67L had larger vesicles compared to Rab7a, consistent with its function of lysosomal fusion and degradation [25,30]. Based on the colocalization results in Figure 3C, Rab7a and Rab7b may share similar localization but may have different functions, as we see Rab7b is preferentially colocalized to the TGN compared to Rab7a. This absence of larger vesicles and the localization of Rab7b may suggest its involvement in cellular recycling/retrograde transport, which is a critical component of the TGN [31]. Our findings are consistent with the literature depicting shared localization of Rab7a and Rab7b to late endosomes and lysosomes with Rab7b preferential to the TGN [32]. These findings suggest the idea that Rab7a and Rab7b may both share the function of lysosomal transport but differ in that Rab7a may be more involved in lysosomal degradation and Rab7b in recycling. This is supported by previous literature demonstrating that the active Rab7a mutant is constrained to lysosomal and late endosomal compartments whilst active Rab7b mutant is also found at the TGN [25]. The presence of Rab7a in the TGN can be supported by its proposed role in the transport of the transferrin receptor [33] and the shared localization of the TGN may be indicative of crosstalk between the Rab7 homologues in the lysosomal and recycling pathways.

### 3.2. Only Rab7b Is Able to Make Splice Isoforms

The Rab7b2/bx8 splice isoforms demonstrated significantly larger and punctate vesicles compared to Rab7b. This is contrary to what we predicted initially, as we expected the spliced-out exons to affect effector/nucleotide binding and therefore vesicle formation. As mentioned previously, the splice isoforms are expressed during early osteoclastogenesis and the presence of vesicles with different morphology and size suggests that the Rab7b splice isoforms have a biological function and this function may differ to Rab7b. We are currently investigating the splice isoforms’ possible functions. Differences in morphology have been shown to be indicative of differential functions, as seen in Rab39a and Rab39b [34]. We are in the process of identifying the function of these vesicles and what roles they may play in subcellular trafficking.

### 3.3. Presence of Vesicles and Nuclear Phenotypes in Rab7a and Rab7b Nucleotide Mutants

The data demonstrated that in Rab7a and Rab7bT22N, and N125/124I nucleotide mutants, there were consistently two populations: cells with vesicles and no vesicles. These vesicles are seen in the published literature but are not discussed [29,32]. The presence of these vesicles could be explained by dimerization, as Rab proteins are able to form dimers in both GTP and GDP-bound states [35]. Unstructured switch regions could lead to dimerization in Rabs [36] and suggest that the mutants can form dimers with the endogenous Rabs to form/associate with the vesicles seen. These dimers may play a role in regulation of the Rab cycle, functioning to establish a cluster of inactive and membrane-bound Rabs [37]. These conserved switch regions may be important in the dimerization of Rab7 and the absence of Switch II in Rab7bx8 suggests alternative pathways are involved in its tethering to the membrane and its presence on vesicles.

While analyzing the cells that presented with a clear vesicle phenotype, we noticed that the distribution of vesicles was not uniform depending on the mutation, as demonstrated in the violin plots (Figures S3B and S4B). In the case of Rab7a and Rab7aQ67L, the distribution was spread out over a larger size range, with the entire range shifted higher for Rab7aQ67L (Figure S3B and Figure 3B). The range for the Rab7aT22N was also spread out, but it appears to consist of two distinct subsets of vesicle sizes (Figure S3B). The Rab7aN125I vesicles collapse into a predominant smaller vesicle population (Figure S3B). The Rab7b vesicle size distribution is broad, but skewed towards smaller vesicles (Figure S4B). This small vesicle population is diminished and shifted towards larger vesicles in Rab7b2, bx8 and N124I expressing cells. Intriguingly, the Rab7bT22N vesicles take on a bimodal distribution that is shifted towards larger vesicles, whereas for Rab7aT22N this bimodal shift was decidedly towards smaller vesicles (compare Figures S3B and S4B). The Rab7bQ67L vesicles behaved like the Rab7aN125I in the sense that the overall population collapsed into a large number of predominant small vesicles, but the average vesicle size for the entire population showed no difference between Rab7b and Rab7bQ67L (Figure 4B and Figure S4B). The bimodal distribution of vesicle sizes could be indicative of lysosomes with different cargos or representing different subcellular compartments (i.e., lysosomes vs. late endosomes). Another possibility is that the different mutants are blocking different steps in the Rab cycle and thus reflect different aspects of vesicle maturation.

When looking at Rab7a and Rab7b localization, we discovered the presence of fluorophores in the nucleus of the mutants. Previous literature has not discussed the presence of Rabs in the nucleus, despite presenting images that demonstrated otherwise [32,38,39]. The nuclear localization was observed in only the splice isoforms and nucleotide mutants, not the WT, with both our GFP and mCherry tags. Other published articles have demonstrated similar nuclear localization of Rabs with other tags [40,41,42,43,44] or untagged endogenous Rabs [45].This suggests that the nuclear localization may be tag-independent and not from introduction/interference of the targeting sequence due to the tag being used. Furthermore, antibody staining of untagged Rab7 demonstrated nuclear localization [46], which may be due to an uncharacterized intrinsic nuclear localization signal or binding to secondary factors to provide the signals for nuclear localization. The preferential nuclear localization of the point mutants or splice isoforms may also be due to interacting with different effectors that are able to import the Rab into the nucleus. We are currently in the process of determining if our Rab fusion proteins are indeed in the nucleus by creating larger Rab7a/b constructs to prevent passive diffusion into the nucleus. In addition, inactive Rab7 has been reported to regulate the function and nuclear localization of Glycogen synthase kinase 3β (GSK3β) [47]. The role of nuclear shuttling of signaling molecules were reported to be significantly reduced in the Rab7 missense mutants with the CMT2B phenotype [48]. This nuclear shuttling regulation may be inhibited in the nucleotide mutants and the CMT2B mutants, thus identifying the sequences involved in nuclear shuttling may be beneficial in targeting and improving the CMT2B phenotypes. As there have been no literature discussing the presence of nuclear localization or export signals in the Rab7 homologues, future research could look into the sequences involved and what roles active or inactive Rab7 may play in the nucleus.

A caveat to our findings is that our Rab7a and Rab7b characterization is limited to HeLa cells. The localization of the Rab7 homologues may vary in various cell models, hence future work will look at their localization and characteristics in other cell lines, particularly during osteoclastogenesis, where the Rab7bx8 isoform is expressed early during differentiation. We predict that overexpression of Rab7bx8 during the entire differentiation program will lead to an observable phenotype.

Our results demonstrate that Rab7b, its nucleotide mutants and splice isoforms behave distinctly from Rab7a in the same experimental set up. Rab7a is associated with human diseases, but no diseases are associated with Rab7b. This possibly suggests that mutations in Rab7b are embryonically lethal or do not have a profound disease phenotype. Future work could look at which pathways Rab7b is specifically involved in. This will require assessing the biological consequences of perturbing Rab7a and Rab7b function in the same experiment, which is currently not done routinely in the field. Further analyses of the Rab7 homologues in alternative cell lines may be informative as to whether their functions are cell-type specific.

The data support our hypothesis that while Rab7a and Rab7b share similar localization, their biological functions are different depending on the cellular context. While the Rab7 homologues are considered as paralogues, one must be wary that they are paralogues with genomic similarity but with divergent functions. We further demonstrate that Rab7b makes at least two different protein splice isoforms that localize to different compartments and could affect effector binding or sequestration. In human diseases, the absence of Rab7a is detrimental, whilst Rab7b absence is beneficial in improving disease phenotypes [13,16,17,18,19]. Understanding the distinct nature between Rab7a and Rab7b is crucial to differentiate between their roles in cellular pathways and human diseases, providing potential targets for future therapies.

## 4. Materials and Methods

### 4.1. Plasmids

Plasmids were previously constructed in the lab and DNA was purified using a GeneJET (Thermo Fisher, Ottawa, ON, Canada) endotoxin-free plasmid maxiprep kit following the manufacturer’s instructions. Plasmids were based on a lentiviral expression system pLV5E using the EF1a promoter or the pCST3 expression plasmid with the CMV IE94 promoter. The different Rab7 proteins were fused to the C-terminus of either mEGFP or mCherry so as not to interfere with the geranylgeranylation of the Rab.

### 4.2. HeLa Cell Transfection

HeLa cells were grown using high glucose DMEM plus 10% FBS at 37 °C in a 5% CO_2_ humidified incubator. Square coverslips were placed in 6-well plates, sterilized with 70% ethanol for 30 min, aspirated, and allowed to dry. The coverslips were then treated with 0.05% gelatin for 5 min before aspiration and allowed to dry. HeLa cells were plated into the 6-well plate and when the cells reached 40–60% confluency, cells were transfected with 2 micrograms of each plasmid using PolyJet transfection reagent (FroggaBio, Vaughan, ON, Canada), following the manufacturer’s instructions. Media were changed the following day, and 48 h after transfection the cells were rinsed once with cold 1xHBSS (GIBCO, 14025-092, Thermo Fischer, Ottawa, ON, Canada), fixed with 4% paraformaldehyde in 1xHBSS pH 7.4 for 15 min at 4 °C, rinsed with 1xHBSS, and mounted on glass slides using a drop of 100% glycerol.

### 4.3. Confocal Microscopy

A Leica Trans Confocal SP8 microscope (Wetzlar, Germany) with 100× objective lens was used to image the cells. Laser powers were adjusted accordingly so that no overexposure was seen on images. These parameters were set for Rab7a and Rab7b, and were kept the same for all the mutants.

### 4.4. Quantitative and Statistical Analysis

Image J (version 1.54f). with plug-in FIJI was used to quantify and compare the average vesicle size indicated by Feret’s diameter. Parameters were adjusted according to the wild type, Rab7b, and kept the same through all mutants. R Studio (R version 4.4.1) was used to generate violin plots of the data analyses. Using SPSS software (Version 29), datasets were analyzed using Factorial Analysis of Variance (Factorial ANOVA) to determine any significant differences between the fluorescent markers (GFP and mCherry). As no significant difference was detected, GFP and mCherry datasets were combined. Data was statistically analyzed using SPSS software, including testing for normality, Kruskal–Wallis tests, and the Mann–Whitney U test for independent samples. Data that were below *p* < 0.05 using the Mann–Whitney U test were considered to be statistically significant. Cells that did not contain bright GFP or mCherry vesicles above the background were not considered to contain vesicles. Cells without vesicles were not included in the quantification of vesicle size and colocalization. A hundred cells were counted to compare the percentage of the population that had vesicles to the percentage of the population with no vesicles. The same one hundred cells were used to compare the percentage that were ‘nuclear and cytoplasmic’ versus ‘cytoplasmic only’.

### 4.5. Clustal Alignment

The Clustal Omega program (https://www.ebi.ac.uk/jdispatcher/msa/clustalo, accessed on 9 March 2025) was used to generate alignments for the Rab7a and Rab7b from a house mouse (*Mus musculus*) [49]. As obtained from the Gene database of NCBI, Rab7a corresponds with NP_033031.2, and Rab7b corresponds with NP_663484.1.

## Data Availability

Data is contained within the article and supplementary material.

## Supplementary Materials

The supporting information can be downloaded at https://www.mdpi.com/article/10.3390/ijms26062610/s1.
