# Supplementary material for: Evidence for Rab7b and Its Splice Isoforms Having Distinct Biological Functions from Rab7a
Source: Int J Mol Sci. 2025 Mar 14;26(6):2610. doi: 10.3390/ijms26062610 (PMC11942325; doi:10.3390/ijms26062610)
Supplement: Supplementary file 1 [file ijms-26-02610-s001.zip › ijms-3431965-supplementary.pdf]

International Journal of Molecular Sciences

MDPI

Supplemental Figures

A

Tests of Between-Subjects Effects

Dependent Variable: Value

Source	Type III Sum of Squares	df	Mean Square	F	Sig.
Corrected Model	3.351[a]	7	.479	3.442	.001
Intercept	114.140	1	114.140	820.553	<.001
FluorescentMarker	.444	1	.444	3.193	.075
Mutant	1.039	1	1.039	7.467	.006
RabType	.268	1	.268	1.923	.166
FluorescentMarker * Mutant	.003	1	.003	.021	.886
FluorescentMarker * RabType	.461	1	.461	3.314	.069
Mutant * RabType	1.242	1	1.242	8.931	.003
FluorescentMarker * Mutant * RabType	.035	1	.035	.253	.615
Error	72.472	521	.139		
Total	277.184	529			
Corrected Total	75.823	528			

a. R Squared = .044 (Adjusted R Squared = .031)

B

Tests of Normality

		Kolmogorov-Smirnov[a]			Shapiro-Wilk		
	Group	Statistic	df	Sig.	Statistic	df	Sig.
Value	Rab7a	.086	70	.200*	.980	70	.337
	Rab7aT22N	.139	70	.002	.948	70	.006
	Rab7aQ67L	.089	70	.200*	.951	70	.008
	Rab7aN125I	.239	33	<.001	.804	33	<.001
	Rab7b	.265	49	<.001	.528	49	<.001
	Rab7bT22N	.198	43	<.001	.885	43	<.001
	Rab7bQ67L	.189	50	<.001	.875	50	<.001
	Rab7bN124I	.100	49	.200*	.925	49	.004
	Rab7b2	.144	48	.014	.905	48	<.001
	Rab7bx8	.233	47	<.001	.695	47	<.001

*. This is a lower bound of the true significance.

a. Lilliefors Significance Correction

Figure S1. A. Factorial ANOVA output from SPSS demonstrating no difference between fluorescent marker type. **B.** Tests of Normality output from SPSS showing non-normal distribution in the Rab7a and Rab7b datasets.

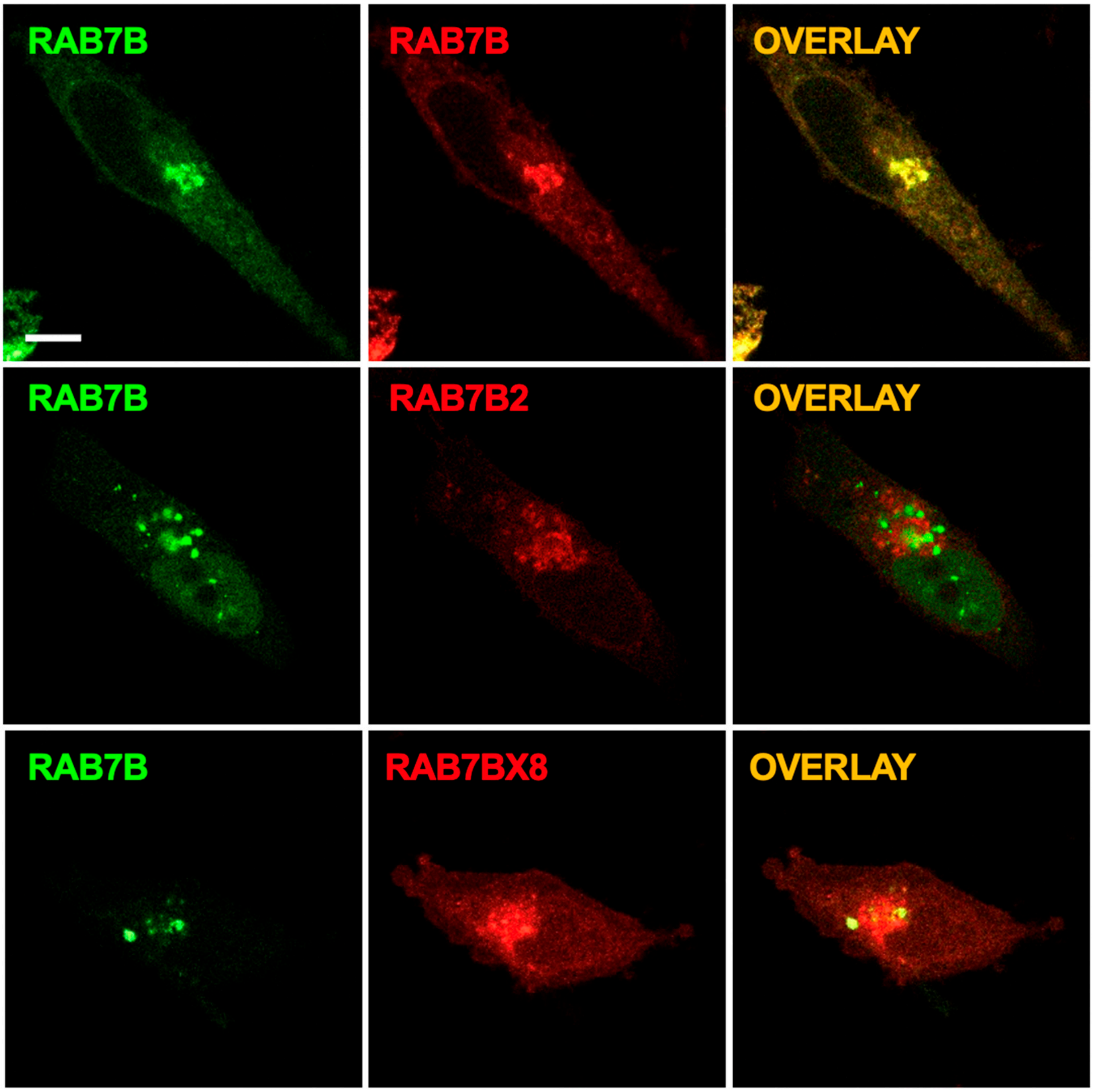

Figure S2. Panel of co-transfected Rab7b in GFP (left column) with its splice isoforms in mCherry (middle column). Overlay (right column) demonstrates any distinct colocalization between the mutants and the Rab7b. Scale bar = 10μm.

A

Test Statistics[a,b]

	Value
Kruskal-Wallis H	209.064
df	3
Asymp. Sig.	<.001

a. Kruskal Wallis Test

b. Grouping Variable: Group

Mann-Whitney Test

Ranks

	Group	N	Mean Rank	Sum of Ranks
Value	Rab7a	70	100.82	7057.50
	Rab7aT22N	70	40.18	2812.50
	Total	140		

Test Statistics[a]

	Value
Mann-Whitney U	327.500
Wilcoxon W	2812.500
Z	-8.846
Asymp. Sig. (2-tailed)	<.001

a. Grouping Variable: Group

Mann-Whitney Test

Ranks

	Group	N	Mean Rank	Sum of Ranks
Value	Rab7a	70	36.23	2536.00
	Rab7aQ67L	70	104.77	7334.00
	Total	140		

Test Statistics[a]

	Value
Mann-Whitney U	51.000
Wilcoxon W	2536.000
Z	-9.998
Asymp. Sig. (2-tailed)	<.001

a. Grouping Variable: Group

Mann-Whitney Test

Ranks

	Group	N	Mean Rank	Sum of Ranks
Value	Rab7a	70	68.46	4792.00
	Rab7aN125I	33	17.09	564.00
	Total	103		

Test Statistics[a]

	Value
Mann-Whitney U	3.000
Wilcoxon W	564.000
Z	-8.142
Asymp. Sig. (2-tailed)	<.001

a. Grouping Variable: Group

B

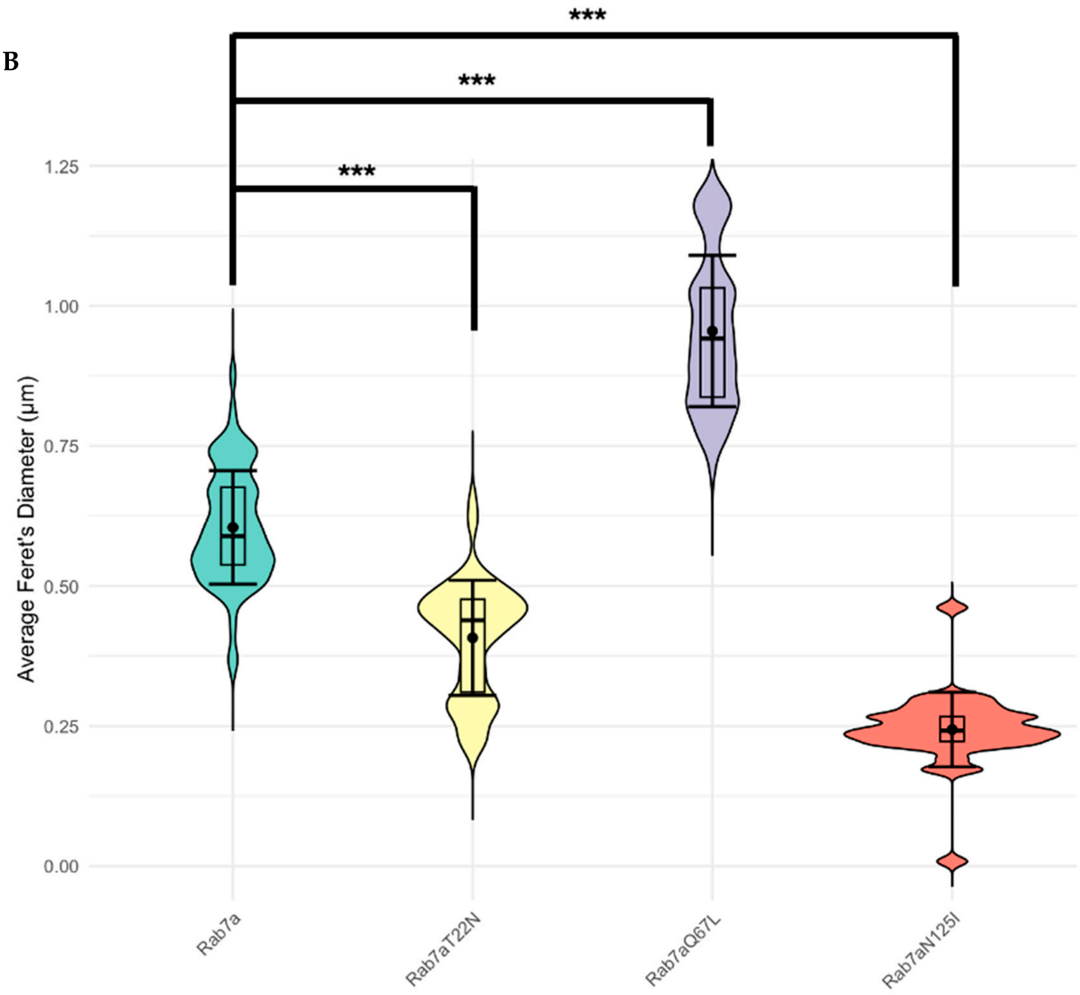

Figure S3. A. Rab7a and its nucleotide mutant's Kruskal-Wallis test and Non-Parametric test output from SPSS. **B.** Violin plot of Rab7a and its nucleotide mutants: including a kernel density plot depicting frequency distribution of data, central box plot and mean indicated by the black point. *** P < 0.001.

A **Test Statistics**[a,b]

	Value
Kruskal-Wallis H	98.776
df	5
Asymp. Sig.	<.001

a. Kruskal Wallis Test

b. Grouping Variable: Group

Mann-Whitney Test

Ranks

	Group	N	Mean Rank	Sum of Ranks
Value	Rab7b	49	34.33	1682.00
	Rab7b2	48	63.98	3071.00
	Total	97		

Test Statistics[a]

	Value
Mann-Whitney U	457.000
Wilcoxon W	1682.000
Z	-5.188
Asymp. Sig. (2-tailed)	<.001

a. Grouping Variable: Group

Mann-Whitney Test

Ranks

	Group	N	Mean Rank	Sum of Ranks
Value	Rab7b	49	28.41	1392.00
	Rab7bx8	47	69.45	3264.00
	Total	96		

Test Statistics[a]

	Value
Mann-Whitney U	167.000
Wilcoxon W	1392.000
Z	-7.216
Asymp. Sig. (2-tailed)	<.001

a. Grouping Variable: Group

Mann-Whitney Test

Ranks

	Group	N	Mean Rank	Sum of Ranks
Value	Rab7b	49	39.24	1923.00
	Rab7bT22N	43	54.77	2355.00
	Total	92		

Test Statistics[a]

	Value
Mann-Whitney U	698.000
Wilcoxon W	1923.000
Z	-2.782
Asymp. Sig. (2-tailed)	.005

a. Grouping Variable: Group

Mann-Whitney Test

Ranks

	Group	N	Mean Rank	Sum of Ranks
Value	Rab7b	49	53.76	2634.00
	Rab7bQ67L	50	46.32	2316.00
	Total	99		

Test Statistics[a]

	Value
Mann-Whitney U	1041.000
Wilcoxon W	2316.000
Z	-1.288
Asymp. Sig. (2-tailed)	.198

a. Grouping Variable: Group

Mann-Whitney Test

Ranks

	Group	N	Mean Rank	Sum of Ranks
Value	Rab7b	49	39.88	1954.00
	Rab7bN124I	49	59.12	2897.00
	Total	98		

Test Statistics[a]

	Value
Mann-Whitney U	729.000
Wilcoxon W	1954.000
Z	-3.350
Asymp. Sig. (2-tailed)	<.001

a. Grouping Variable: Group

B

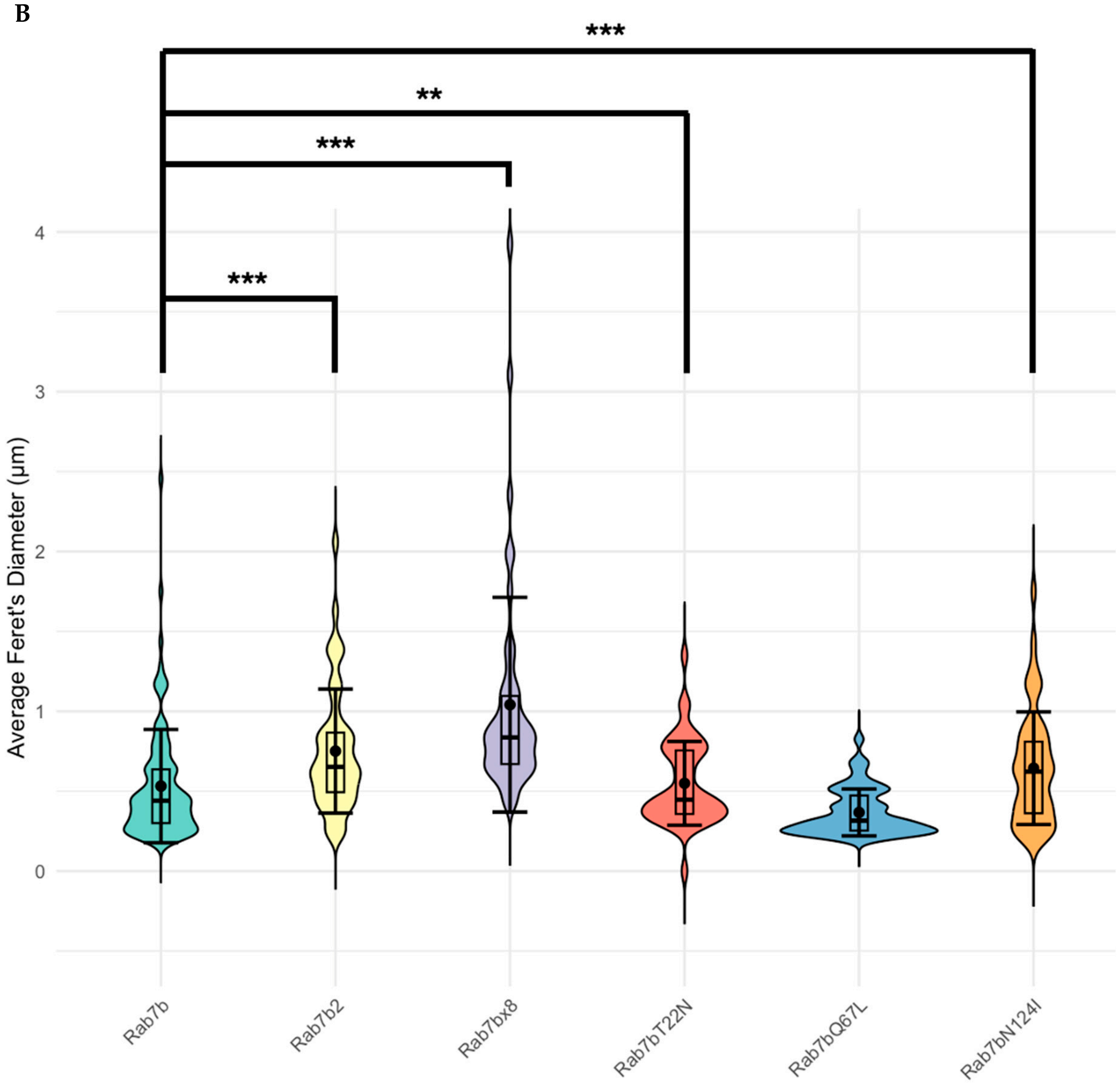

Figure S4. A. Rab7b, Rab7b splice isoforms and its nucleotide mutant's statistical Kruskal-Wallis test and Non-Parametric test output from SPSS. **B.** Violin plot of Rab7b, Rab7b splice isoforms and its nucleotide mutants: including a kernel density plot depicting frequency distribution of data, central box plot and mean indicated by the black point. ** $P < 0.01$. *** $P < 0.001$.

Test Statistics[a,b]

	Value
Kruskal-Wallis H	252.990
df	7
Asymp. Sig.	<.001

a. Kruskal Wallis Test

b. Grouping Variable: Group

Mann-Whitney Test

Ranks

	Group	N	Mean Rank	Sum of Ranks
Value	Rab7a	70	78.10	5467.00
	Rab7b	49	34.14	1673.00
	Total	119		

Test Statistics[a]

	Value
Mann-Whitney U	448.000
Wilcoxon W	1673.000
Z	-6.841
Asymp. Sig. (2-tailed)	<.001

a. Grouping Variable: Group

Mann-Whitney Test

Ranks

	Group	N	Mean Rank	Sum of Ranks
Value	Rab7aT22N	70	50.81	3557.00
	Rab7bT22N	43	67.07	2884.00
	Total	113		

Test Statistics[a]

	Value
Mann-Whitney U	1072.000
Wilcoxon W	3557.000
Z	-2.561
Asymp. Sig. (2-tailed)	.010

a. Grouping Variable: Group

Mann-Whitney Test

Ranks

	Group	N	Mean Rank	Sum of Ranks
Value	Rab7aQ67L	70	85.31	5972.00
	Rab7bQ67L	50	25.76	1288.00
	Total	120		

Test Statistics[a]

	Value
Mann-Whitney U	13.000
Wilcoxon W	1288.000
Z	-9.246
Asymp. Sig. (2-tailed)	<.001

a. Grouping Variable: Group

Mann-Whitney Test

Ranks

	Group	N	Mean Rank	Sum of Ranks
Value	Rab7aN125I	33	21.88	722.00
	Rab7bN124I	49	54.71	2681.00
	Total	82		

Test Statistics[a]

	Value
Mann-Whitney U	161.000
Wilcoxon W	722.000
Z	-6.123
Asymp. Sig. (2-tailed)	<.001

a. Grouping Variable: Group

Figure S5. Kruskal-Wallis test and non-parametric tests output from SPSS demonstrating significant differences between Rab7a and Rab7b nucleotide mutants.

Tests of Normality

	Kolmogorov-Smirnov[a]			Shapiro-Wilk		
	Statistic	df	Sig.	Statistic	df	Sig.
Values	.077	60	.200*	.975	60	.243

*. This is a lower bound of the true significance.

a. Lilliefors Significance Correction

Group Statistics

	Group	N	Mean	Std. Deviation	Std. Error Mean
Values	Rab7a and TGN	30	.5300	.16314	.02978
	Rab7b and TGN	30	.6173	.12998	.02373

Independent Samples Test

		Levene's Test for Equality of Variances		t-test for Equality of Means							
						Significance		Mean Difference	Std. Error Difference	95% Confidence Interval of the Difference	
		F	Sig.	t	df	One-Sided p	Two-Sided p			Lower	Upper
Values	Equal variances assumed	1.896	.174	-2.293	58	.013	.025	-.08733	.03808	-.16357	-.01110
	Equal variances not assumed			-2.293	55.244	.013	.026	-.08733	.03808	-.16365	-.01102

Independent Samples Effect Sizes

		Standardizer[a]	Point Estimate	95% Confidence Interval	
				Lower	Upper
Values	Cohen's d	.14750	-.592	-1.107	-.072
	Hedges' correction	.14944	-.584	-1.093	-.071
	Glass's delta	.12998	-.672	-1.201	-.132

a. The denominator used in estimating the effect sizes.
Cohen's d uses the pooled standard deviation.
Hedges' correction uses the pooled standard deviation, plus a correction factor.
Glass's delta uses the sample standard deviation of the control (i.e., the ...

Figure S6. Tests of normality and t-tests output from SPSS demonstrating significant differences between colocalization of TGN with Rab7a compared to Rab7b.
